# The effect of contextualised match variables on the metabolic power of elite soccer players during English Premier League match-play

**DOI:** 10.5114/biolsport.2026.156783

**Published:** 2025-12-04

**Authors:** Ryland Morgans, Rafael Oliveira, Mauro Mandorino, Ben Ryan, Piotr Zmijewski, Toni Modric, Jose Eduardo Teixeira, Alexandre Moreira

**Affiliations:** 1 School of Sport and Health Sciences, Cardiff Metropolitan University, Cardiff, UK; 2 Brentford FC Football Research Centre, Brentford FC, London, UK; 3 Research Centre in Sports Sciences, Health and Human Development, (CIDESD), Santarém Polytechnic University, 2040-413 Rio Maior, Portugal; 4 Santarém Polytechnic University, School of Sport, 2040-413 Rio Maior, Portugal; 5 Performance and Analytics Department, Parma Calcio 1913, 43121 Parma, Italy; 6 Department of Movement, Human and Health Sciences, University of Rome “Foro Italico”, 00135 Rome, Italy; 7 Jozef Pilsudski University of Physical Education in Warsaw, 00-809 Warsaw, Poland; 8 Research and Development Center Legia Lab, Legia Warszawa, Poland; 9 Faculty of Kinesiology, University of Split, Split, Croatia; 10 High Performance Sport Center, Croatian Olympic Committee, Zagreb, Croatia; 11 Department of Sports Sciences, Polytechnic of Guarda, Guarda, Portugal; 12 Department of Sports Sciences, Polytechnic of Cávado and Ave, Guimarães, Portugal; 13 SPRINT-Sport Physical Activity and Health Research & Innovation Center, Guarda, Portugal; 14 Department of Sport, School of Physical Education and Sport, University of São Paulo, São Paulo, Brazil

**Keywords:** Match-play, Metabolic power, Playing position, Match period, Opponent ranking, Team formation, Match status, Location, Optical tracking, Football, Soccer

## Abstract

This study examined the effect of contextualised match variables on metabolic power of elite soccer players during English Premier League (EPL) match-play across four seasons (2021/22–2024/25), comparing in-possession (MP_IP_) and out-of-possession (MP_OP_) phases. Match data from 31 male professional soccer players retrieved from 152 regular-season EPL competitive matches was obtained via an optical tracking system and analysed with decision tree regression models. The MP_IP_ model demonstrated strong predictive accuracy (RMSE = 1.54; MPE = 2.04%), identifying playing position as the dominant predictor (88% of total feature importance). Defenders exhibited the lowest MP_IP_, while forwards in a 3-5-2 team formation showed the highest values (MP_IP_ = 15.7 W · kg^−1^). Conversely, the lowest MP_IP_ values (MP_IP_ = 12.1 W · kg^−1^) were observed when the study team did not employ a 3-5-2 team formation and faced opponents with a ranking difference of less than eight positions. The MP_OP_ model also demonstrated robust predictive performance (RMSE = 1.59; MPE = 1.30%). Playing position was the most influential factor (44% of total feature importance), with midfielders displaying higher MP_OP_. Forwards had the lowest MP_OP_ in the second half, while the highest values (MP_OP_ = 17.1 W · kg^−1^) were observed for midfielders when the team was losing in a 3-5-2 formation. These findings confirm that positional role is the principal determinant of metabolic power in EPL match-play, with midfielders particularly exposed to elevated demands out-of-possession. Tactical structure, match status, and match period further modulate metabolic load, highlighting the need to consider context-specific training strategies.

## INTRODUCTION

Match demands in the English Premier League (EPL) are complex [1] with numerous influences on player running performances [2–9]. Contextual variables including possession, positional differences, opposition ranking, team formation, and match location have recently been found to influence match running performance [2, 5, 7, 8].

Elite EPL soccer requires players to perform increasingly more highintensity activities throughout training and match-play [1, 3, 5, 9]. Analysis of running demands in elite soccer has mainly focused on distance and speed-based measurements [10]. However, these analyses may underestimate the intricate, high-energy activities such as accelerations (ACC) and decelerations (DEC) and the subsequent metabolic power output [11], which are essential in elite soccer performance [12]. Metabolic power has been previously proposed as another indicator to quantify physical activity and is calculated from the energy cost and the speed activities performed during physical performance [13]. The principle of metabolic power suggests that higher values are present immediately following the execution of acceleration-type actions compared to constant running [13]. Thus, metabolic power has been suggested and applied as a practical measure to more accurately quantify players’ energy cost [11]. As explosive actions during elite soccer actions continue to be examined [1–3, 5, 11, 13], utilising metabolic power as a key marker may enhance the analysis of the physical demands of intermittent characteristics.

High-intensity actions such as accelerating and decelerating have recently been shown to significantly influence decisive moments of the match [15, 16], thus understanding the individual metabolic power output of players may enhance a tailored approach to training, match preparation, recovery and ultimately team success. Morgans et al. [1] highlighted that these high-intensity actions (ACC and DEC) expressed as high-power output are two to three times larger than those based only on running speed [13], irrespective of playing position [14–17]. Therefore, it appears crucial for practitioners to profile such explosive actions and the subsequent metabolic power profiles throughout a match, across a season and multiple seasons so that players can be prepared to cope with the physiological and mechanical demands of elite match-play [2–5].

Players’ physical match demands, and subsequent metabolic power output, are significantly influenced by match context [1, 8, 9, 18]. It has been explored that contextualised factors such as opponent standard, match location, and ball possession are highly influential on players’ match performance, including sprint, high-speed running, high-intensity running, sprinting and ACC and DEC performance [1, 7, 8, 18, 19]. For example, Morgans et al. [2] showed that players performed more ACC and DEC when playing against higher-standard opponents. Additionally, Morgans et al. [8] found that players exhibited higher physical performance, including ACC and high-intensity actions at home compared to away matches and suggested the influences of different playing formations and ball possession on the physical demands of players. Therefore, an understanding of the influences of contextual factors on players’ physical match demands is essential to truly evaluate soccer performance [20]. Although, metabolic power profiling was not considered as part of these studies.

Players need to execute explosive actions, while under pressure during in- and out-of-possession [8, 18, 21]. Therefore, the context in which these high-intensity match actions occur is vital to fully understand how to optimally prepare players for positional demands and the game [1–8, 16–21]. For example, positional differences playing against top, middle and bottom teams at home or away have recently been highlighted [1, 3, 5, 7, 8], however, these studies did not examine the influence of contextual variables on metabolic power output, limiting the generalisation of the results to other teams and leagues. Therefore, further research is warranted.

Currently there is limited evidence examining contextualised metabolic power profiles during elite-level soccer match-play in relation to possession, positional differences, match period, opponent ranking, team formation, match status and location, thus the current study would seem to offer real-world practical meaning. The aims of this study were, firstly, to compare the average metabolic power when in- and out-of-possession during official EPL match-play over four consecutive seasons; and secondly, to examine any differences considering playing position, match period, opponent ranking, team formation, match status and location. The study hypothesis was that positional differences of average metabolic power would be evident, with an increase when out-of-possession during EPL match-play and that match period, opponent ranking and match status would also influence metabolic power outputs.

## MATERIALS AND METHODS

### Study Design

A retrospective study was performed analysing EPL match data from the 2021/2022 to 2024/2025 seasons for 31 male professional soccer players. The EPL comprises of 38 matches, 19 home and 19 away across a 10-month season, commencing in August and completing in May. Data were collected via an optical tracking system from 20 EPL stadiums.

This study utilised a four-year longitudinal study design. A nonprobabilistic sampling protocol was engaged to recruit the participants. The focus of the study was on players metabolic power while in- and out-of-possession during competitive EPL match-play. During the observational seasons 2021/2022 to 2024/2025, consistent player monitoring approaches were applied without any intrusion from the researchers [3].

### Participants

Thirty-one professional first-team squad outfield soccer players from an EPL club were part of the study (age 24.6 ± 5.4 years, weight 76.6 ± 6.9 kg, height 1.79 ± 0.09 m). The EPL team adopted a 4-3-3 or 3-5-2 formation and implemented a hybrid model of possession that included a combination of build-up and direct-play strategies [1, 3, 18]. Furthermore, when out-of-possession a mixture of high-press and mid-block (a narrow and compact team shape defending the middle third of the pitch) strategies were employed [1, 18].

The research inclusion criteria have been recently employed [1–8, 18] and were: (i) named in the first-team squad at the start of all study seasons, (ii) played in at least 80% of matches, (iii) only completed official team training during the study period, and (iv) completed at least 90-minutes of match-play. Additionally, the exclusion criteria for the study included: (i) long-term injury (three months or longer), (ii) joining the team late (following the completion of pre-season) in any of the study seasons, (iii) lack of full, complete match data [1–8, 18], and (iv) goalkeepers, due to the variations in physical demands.

Players were categorised as: defenders (n = 252), midfielders (n = 296), and forwards (n = 96). Players were classified according to the main position, defined as the one occupied for >50 mins of match time. Considering the high variability of metabolic power profiles, only players who completed the full match were considered in the analysis [26].

All data collected resulted from normal analytical procedures regarding player monitoring over the competitive season [1–8, 18], nevertheless, written informed consent was obtained from all participants. The study was conducted according to the requirements of the Declaration of Helsinki and was approved by the local Ethics Committee of Cardiff Metropolitan University (Sta-9172) and the club from which the participants volunteered [27]. To ensure confidentiality, all data were anonymised prior to analysis.

### Match Data Collection

Data were collected during all (n = 152) regular-season EPL competitive matches played by the examined team across the four study seasons (2021/2022 to 2024/2025).

League match data across the study seasons were recorded and analysed via the optical tracking system Second Spectrum to report physical performance data. Second Spectrum has been validated by the FIFA Quality Programme to meet industry standards [28]. Data were collected via semi-automated HD cameras positioned around the stadium with a sampling frequency of 25 Hz.

Average metabolic power ([W · kg^−1^]) was selected based on previous publications [1, 18, 22, 23]. Data were filtered according to the theoretical model based on an energetic approach where the energy cost of ACC and DEC plays a central role [11, 22, 24, 25]. Average metabolic power was also quantified when in-possession (MP_IP_) and when opposition in-possession (MP_OP_).

Metabolic power output included in the analysis was calculated utilising previous research in estimating the energy cost (EC) of accelerated and decelerated running [11]. Average metabolic power was determined for every individual frame using instantaneous acceleration and speed observed in that frame based on the early work by Osgnach et al. [11] and further developed by di Prampero et al. [13] that reported accelerated running on a flat terrain is energetically equivalent to uphill running at a constant speed. During sprinting, the body leans forward forming an angle (a) with the terrain [11, 13]. Accelerated running can be considered equivalent to running at a constant speed up an ‘‘equivalent slope’’ (ES) where:


ES=tan(90-α) [11, 13]

In addition, the average force exerted by the active muscles during sprinting is greater than the athlete’s body weight and represents an overload imposed on the athlete by the acceleration itself known as the ‘‘equivalent mass’’ (EM) [11, 13].

Metabolic power (P) was then calculated multiplying EC by running speed (v):


P=EX×v

Therefore, once speed and acceleration are known, the metabolic power output of each player in any given moment can be easily obtained [11, 13].

Each match was classified based on the ranking difference between the study team and opponent at that specific point in the season [1, 18]. This ranking difference was treated as a continuous variable, reflecting the number of positions separating the two teams in the league standings [1, 18]. A positive value indicated that, at that moment in the season, the team held a higher (better) rank than its opponent, whereas a negative value signified that the team was ranked lower (worse) than its opponent [1, 18].

The Second Spectrum match data were processed directly using the Python programme (v 3.11), through the Spyder scientific development environment (https://www.spyder-ide.org/). Publishing the exact algorithms used to determine the examined metrics was not possible due to the intellectual property rights of the technological commercial entities [29]. Therefore, the specific conversion and filtering algorithms utilised in these systems were not available.

### Statistical Analysis

The statistical analysis aimed to investigate the influence of contextual match variables on players’ average metabolic power output, which was measured separately for match-play in-possession (MP_IP_) and out-of-possession (MP_OP_). Therefore, separate Decision Tree Regressor (DTR) models were selected utilising the two continuous variables of MP_IP_ and MP_OP_ as dependent variables. Among the contextual match variables, the following features were selected: playing position: defenders, midfielders, and forwards; temporal match period: first and second half; ranking difference: difference in ranking between the team considered and opponent team at kick-off; team formation: the structure of the team (3-5-2 [team], 4-2-3-1 [team], 4-3-3 [team], 4-4-2 [team]) and the opponent’s team formation (3-5-2 [opponent], 4-2-3-1 [opponent], 4-3-3 [opponent], 4-4-2 [opponent]); match status: winning, drawing, losing; and match location: home and away.

All categorical variables were previously one-hot encoded and verified for multicollinearity to ensure orthogonality in the regression tree splits. The dataset, prior to the model training, was partitioned into training (80%) and testing (20%) subsets via stratified random sampling. No additional transformations (e.g., scaling or normalisation) were required to the non-parametric nature of the decision tree. Decision Tree Regressor models were selected based on the capacity to capture non-linear relationships, model high-order interactions, and generate interpretable decision rules [30, 31]. Decision Trees were constrained to a maximum depth of three levels, balancing model complexity with interpretability and minimising the risk of overfitting. Model fitting was conducted with recursive binary splitting based on the minimisation of the mean squared error. Model generalisation was assessed on the held-out test using root mean squared error (RMSE) and mean percentage error (MPE), which allowed for capturing both absolute and proportional deviation between observed and predicted values.

Following model fitting, feature importance scores were extracted to quantify the relative contribution of each predictor to the tree’s explanatory power. All the statistical procedures were implemented using the Python programme (v 3.11), leveraging the pandas, scikitlearn, matplotlib, and numpy libraries.

## Results

Descriptive statistics for categorical variables are reported as mean ± standard deviation in Table 1.

**TABLE 1:** Descriptive statistics of the average metabolic power ([W · kg^−^^1^]) in-possession (MP_IP_) and out-of-possession phase (MP_OP_) by playing position, temporal match period, team formation, match status, and match location. Values are expressed as mean ± standard deviation (SD).

Variables	Sub-groups	MP_IP_	MP_OP_
Playing Position	Defenders	12.49 ± 1.54	14.80 ± 1.44
	Midfielders	14.52 ± 1.38	15.70 ± 1.81
	Forwards	14.84 ± 1.91	13.28 ± 1.81

Temporal Match Period	First half	13.75 ± 1.83	15.09 ± 1.79
	Second half	13.33 ± 1.90	14.68 ± 1.87

Team Formation	3-4-3 [team]	13.37 ± 1.83	14.72 ± 1.69
	3-5-2 [team]	13.96 ± 2.02	15.09 ± 1.90
	4-2-3-1 [team]	13.43 ± 1.78	14.81 ± 1.78
	4-3-3 [team]	13.95 ± 1.76	14.69 ± 1.61
	4-4-2 [team]	13.67 ± 1.73	14.81 ± 1.58
	3-4-3 [opponent]	13.56 ± 1.68	14.97 ± 1.81
	3-5-2 [opponent]	13.51 ± 1.90	15.20 ± 1.84
	4-2-3-1 [opponent]	13.65 ± 1.73	14.82 ± 1.84
	4-3-3 [opponent]	13.84 ± 1.73	15.32 ± 1.86
	4-4-2 [opponent]	13.69 ± 1.75	15.25 ± 1.75

Match Status	Winning	13.73 ± 1.82	15.08 ± 1.81
	Drawing	13.42 ± 1.77	14.76 ± 1.80
	Losing	13.72 ± 1.95	15.06 ± 1.84

Match Location	Home	13.69 ± 1.87	14.93 ± 1.81
	Away	13.58 ± 1.84	15.04 ± 1.83

### MP_IP_ Model

**FIG. 1:**
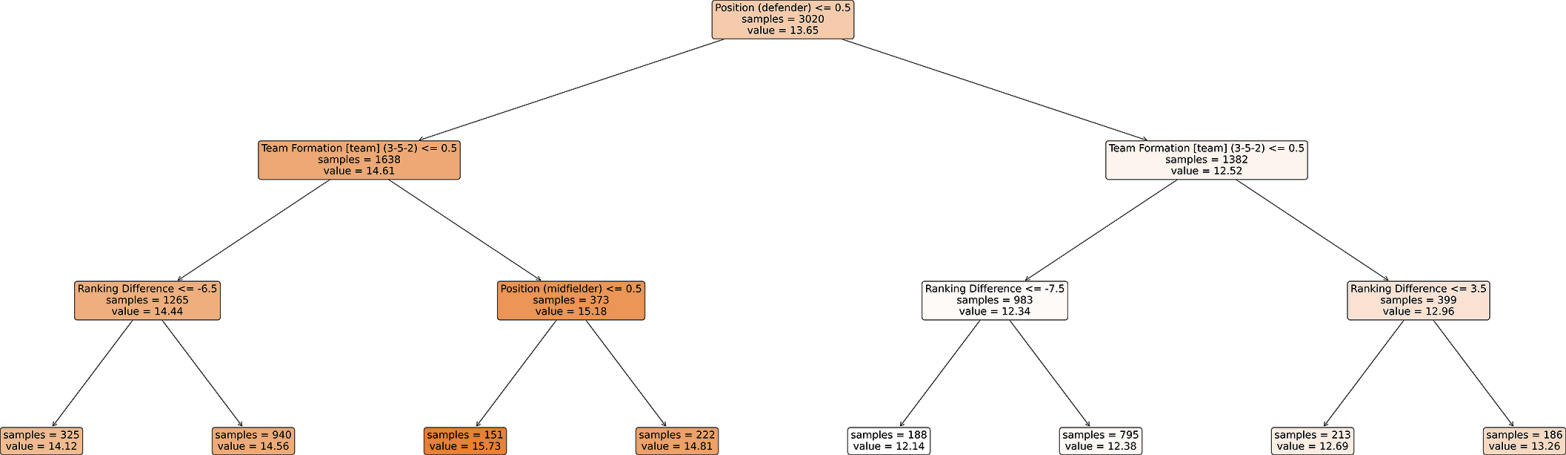
The structure of the Decision Tree Regressor (DTR) for the match-play in-possession (MP_IP_) model.

The MP_IP_ model demonstrated good predictive accuracy, with a RMSE of 1.54 and a MPE of 2.04% on the held-out test set. Feature importance analysis revealed that playing position was the dominant predictor. Specifically, the categorical variable playing position (defender), indicating whether the player was classified as a defender, was selected as the root node in the decision tree and accounted for 88% of the total feature importance (Table 2). Players classified as defenders displayed a lower average metabolic power during the possession phase compared with all other positions. The second most influential predictor was team formation (3-5-2 [team]), which appeared in both the left and right subtrees and explained 7.2% of the variance. In the left subtree, the highest MP values were observed for forwards playing within a 3-5-2 formation (MP = 15.7). In the right subtree, the lowest MP values were recorded when the team formation differed from 3-5-2 and when playing against opponents with a ranking difference lower than eight positions (MP = 12.14). The structure of the DTR for the MP_IP_ model is presented in Figure 1.

**TABLE 2:** Feature importance analysis for the two decision tree regression models built to predict the average metabolic power inpossession (MP_IP_) and out-of-possession phase (MP_OP_).

Variables	Importance % (MP_IP_)	Importance % (MP_OP_)
Position (Defenders)	88	28
Position (Midfielders)	2	44
Team Formation [Team] (3-5-2)	8	12
Ranking difference	2	9
Temporal Match Period	0	5
Match Status (Losing)	0	2

### MP_OP_ Model

The MP_OP_ model also demonstrated robust predictive performance, with an RMSE of 1.59 and an MPE of 1.30%. In this model, playing position remained the most influential variable, accounting for 44% of the total feature importance. Midfielders exhibited higher average metabolic power during MP_OP_ compared to all other positions. The decision tree revealed distinct patterns across subtrees: (1) Left subtree: the lowest MP values were recorded, particularly for forwards in the second half of matches, indicating reduced physical demands in this context, (2) Right subtree: the highest MP values were associated with midfielders when the study team was losing in a 3-5-2 team formation (MP_OP_ = 17.08), highlighting the combined effect of positional role, tactical organisation, and match status.

**FIG. 2:**
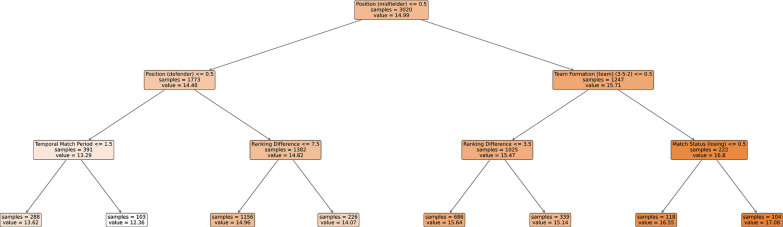
The structure of the Decision Tree Regressor (DTR) for the match-play out-of-possession (MP_OP_) model.

Unlike the MP_IP_ model, the MP_OP_ model’s predictions were influenced by a broader range of variables (Table 2). The structure of the DTR for the MP_OP_ model is illustrated in Figure 2.

## DISCUSSION

This study aimed to compare metabolic power when in- and out-ofpossession during official EPL match-play over four consecutive seasons; and secondly, to examine any differences considering playing position, match period, opponent ranking, team formation, match status and location. The main finding of this study was that playing position was the strongest predictor of metabolic power profiling during EPL match-play in both models tested. Specifically, during MP_IP_, defenders had lower metabolic power compared to all other positions. Moreover, during MP_OP_, midfielders showed the highest metabolic power, especially when the team was losing in a 3-5-2 team formation. Additionally, the MP_OP_ model was also influenced by a broader range of contextual variables (team formation, match period and match status) than the MP_IP_ model.

In support of previous research [32], playing position was the most relevant contextual factor. Such findings were recently highlighted in a study conducted in the EPL that showed the influence of this contextual variable in ACC when the study team was in-possession and DEC when in- and out-of-possession [1]. Specifically, the study demonstrated that defenders had the lowest number of DEC when out-ofpossession, while midfielders and defenders had the lowest numbers of both ACC and DEC when in-possession [1]. Such results were partially supported by the findings of the present study, since defenders also showed the lowest MP_IP_, although forwards showed the lowest values when out-of-possession which contrasts the previous study [1]. This can partially be explained by differences in tactical roles where defenders and midfielders aim to fulfil positioning to intercept opponent forward passes or prevent quick, direct counter-attack transitional play, which typically results in less MP_OP_ when compared to other positions, such as attackers [33, 34].

Furthermore, midfielders showed the highest MP_OP_, especially when the study team was losing and the team formation was 3-5-2. Nonetheless, previous research showed that team formation and possession can have a significant impact on total distance, highspeed running, and high metabolic load distance in the EPL [8]. In particular, Morgans et al. [8] reported higher values for the 3-5-2 compared to the 4-3-3 team formation, which tends to support the findings of the present study. In addition, a previous Morgans et al. [5] study conducted in EPL found that players covered significantly more m/min and a higher number of DEC when playing against the top six compared to mid-table or bottom six teams, however no differences in external match load when analysing match outcome were displayed, which was not similar to the present findings.

Based on existing literature [20, 21, 32], the study hypothesis was that positional differences of metabolic power would be evident, with an increase when out-of-possession during EPL match-play. It is relevant to highlight that the values shown in the present study were superior to those displayed by the German Bundesliga [32] which supports previous findings that the EPL produces higher demands than other European leagues (e.g. French Ligue 1) [3].

The MP_OP_ model was notably influenced by position, specifically defenders recorded the lowest metabolic power compared with MP_IP_. This result suggests greater complexity of match running demands during the non-possession phase. Such a finding is in line with previous research that found higher values of distance covered (+31%) and at high-speed (+30%) during out-of-possession compared with in-possession [35]. This result provides practitioners with detailed information to support the design of position-specific drills and the differences of these actions while in- and out-of-possession [35].

### Practical Applications

The findings of this study offer several practical applications for coaches, analysts, and sports scientists involved in elite soccer, particularly in the EPL. The preparation for higher demands when inpossession should specifically focus on midfielders and forwards, while during out-of-possession all positions should be considered. Such scenarios could potentially be trained and developed through various sided-games (small and medium) and position-specific drills. Moreover, for low-possession teams, a higher emphasis on out-ofpossession drills which prioritise higher physical fitness by improving the capacity of players to perform repeated bouts of explosive actions (e.g. closing down, high pressing) could be beneficial [35]. As previously recommended [35], a defensive tactical strategy that focuses on high-intensity pressing to regain possession quickly and minimise the overall physical demands while reducing the metabolic power of match-play (by maximising possession) could be a strategy to consider. Specifically, forwards and midfielders may emphasise position-specific drills that physically enhance explosive speed, agility, and sharp cuts to improve accelerations/decelerations through various attacking patterns that complete with an offensive action (for instance, a line-breaking final pass from midfield to the forwards, a shot at goal or cross from wide area), subsequently, integrating MP_IP_ and physical demands. Furthermore, several types of sidedgames (with varying pitch sizes) can be utilised. For instance, players are repeatedly forced to execute quick transitions from defending to receiving the ball to create attacking options, while defenders and midfielders may focus on tactical awareness and positioning drills, incorporating scenarios that simulate intercepting passes and maintaining defensive shape during out-of-possession phases [36]. Considering the aims of the present study, Morgans et al. [37] provided detailed information examining small-sided-games in a recent publication. Finally, considering the high physical demands associated with such position-specific training, the practical recommendation from this study would suggest that these types of drills and sessions would be most suitable on match-day (MD) -4 (four days prior to the next competitive match) and MD-3 training days of a microcycle.

### Limitations and Future Research Direction

Despite the novel approach in the present study that highlighted playing position as the main contextual factor, only three positions were categorised and examined. Moreover, average metabolic power could have been analysed relatively, while additional metrics such as high metabolic power distance (HMPD > 25 ([W · kg^−1^]), number of power events (n) and average power recovery between power events ([W · kg^−1^]), which would provide greater granularity of the results, could have also been examined as absolute and relative values. In addition, other related metrics such as accelerations/decelerations and energy expenditure would provide further insights. Future research should consider examining other playing positions and player status (starters versus non-starters) to potentially display varying findings. For instance, wide defenders, midfielders, and attackers/forwards can be separated and considered. While future studies could also analyse the differences between starting and nonstarting players, allowing training drills to theoretically be designed to ensure non-starting players produce a greater impact during limited match-play minutes.

Furthermore, the present study could be statistically replicated with common external load measures (e.g., high-speed running, sprinting, ACC and DEC) to understand if the findings are in line with those reported previously examining metabolic power [35] and the potential benefits of utilising open-source software and ensuring this information is freely available will enhance future research and outcomes for practitioners. Finally, the replication of the present study in different contexts is warranted (e.g., different countries, leagues, levels, gender, and young teams).

## CONCLUSIONS

This study identified that distinct contextual factors influence metabolic power during EPL match-play. Specifically, playing position was the strongest predictor of metabolic power. Defenders showed lower metabolic power during MP_IP_ compared to all other positions. Midfielders showed the highest metabolic power during MP_OP_, especially when losing in a 3-5-2 team formation. Lastly, during MP_OP_, metabolic power was influenced by a broader range of contextual variables (team formation, match period and match status) than the MP_IP_ model, suggesting greater complexity during non-possession phases.
